# Rapid on-site dual optical system to measure specific reactive oxygen species (O_2_^-•^ and OCl^-^) in a tiny droplet of whole blood

**DOI:** 10.1371/journal.pone.0200573

**Published:** 2018-08-01

**Authors:** Kimiko Kazumura, Kozo Takeuchi, Akiko Hara, Toshiyuki Miwa, Masaki Hattori, Yuqiu Wu, Naokazu Morishita, Hiroshi Tsuchiya, Toshihiko Osawa

**Affiliations:** 1 Central Research Laboratory, Hamamatsu Photonics K.K., Shizuoka, Japan; 2 Global Strategic Challenge Center, Hamamatsu Photonics K.K., Shizuoka, Japan; 3 Department of Research and Development, Healthcare Systems Co. Ltd., Aichi, Japan; 4 Electron Tube Division, Hamamatsu Photonics K.K., Shizuoka, Japan; 5 Department of Health and Nutrition, Faculty of Psychological and Physical Science, Aichi Gakuin University, Aichi, Japan; The University of Tokyo, JAPAN

## Abstract

Oxidative stress has been implicated in various disorders and controlling it would be important for healthy life. We have developed a new optical system for easily and accurately measuring oxidative stress in whole blood. It is optimized for simultaneously detecting reactive oxygen species (ROS) and highly reactive ROS (hROS), elicited mostly by white blood cells in a few microliters of blood. Results obtained by using this system show at least four important findings. 1) chemiluminescence of MCLA was confirmed to be attributable to O_2_^-•^. 2) PMA-stimulated cells released O_2_^-•^ longer and more slowly than fMLP-stimulated ones. 3) fluorescence produced by APF oxidation was confirmed to be attributable to hROS, mostly OCl^-^, produced by myeloperoxidase. 4) the generation of OCl^-^ was found to be a slower process than the O_2_^-•^ generation. We also conducted pilot studies of oxidative stress in healthy volunteers.

## Introduction

Oxidative stress is the term describing an imbalance between oxidant production, potentially leading to oxidative injury, and antioxidant capacity of the cells, bodily fluids and tissues [1,2]. Afterwards, the definition of oxidative stress has been re-defined as “an imbalance between oxidants and antioxidants in favor of the oxidants, leading to a disruption of the redox signaling and control, and/or molecular damage” [3]. Oxidative stress has been implicated in various diseases such as cardiovascular diseases, neurodegenerative disorders, cancer, renal and lung diseases [4,5]. Therefore, it is important to monitor thus produced oxidants, collectively called reactive oxygen species (ROS), at an early stage for preventing such diseases.

ROS are thought to be formed as an unwanted byproduct of aerobic metabolism but can also be produced at elevated rates under pathophysiological conditions [1–5]. In fact, all phagocytic cells, including white blood cells (WBC) have a well-characterized superoxide radical (O_2_^-•^) generating plasma membrane oxidase capable of producing the large amounts of ROS required for its function in host defense [6]. O_2_^-•^ produced by WBC is immediately converted to hydrogen peroxide (H_2_O_2_) by spontaneous disproportionation reactions [6]. H_2_O_2_ is then enzymatically converted to more reactive oxygen radicals including hypochlorite ions (OCl-) by myeloperoxidase (MPO) [6,7].

We noticed that both O_2_^-•^ and OCl- can be measured simultaneously by modifying the previously developed system that can monitor generated O_2_^-•^ and intracellular Ca^2+^ concentrations [8–11], which lead us to develop a new system. The new system uses the same chemiluminescence (CL) probe of MCLA (CL_MCLA_) for O_2_^-•^ as before [12,13] while OCl- is monitored as fluorescence (FL) by using APF (FL_APF_) [14].

Here we report about the improvements of the new system optimized for monitoring ROS in a small amount of whole blood (1.5~3μl). Among them, we would like to point out that the measuring liquid samples are formed as confined suspensions placed on a special glass slide, which enables optical path lengths short enough to suppress blood component light absorptions. The system performs the subsequent procedures semi-automatically after introduction of whole blood into it. We also show that the system was successfully applied to a pilot study of the effects of diet and exercise on the oxidative stress of volunteers.

## Materials and methods

### Reagents

Dimethyl sulfoxide (DMSO), N-formyl-methionyl-leucyl-phenylalanine (fMLP), 4-aminobenzoic acid hydrazide (ABAH), phorbol 12-myristate 13-acetate (PMA), hypoxanthine and xanthine oxidase were purchased from Sigma-Aldrich Japan (Tokyo, Japan). 2-Methyl-6-(4-methoxyphenyl)-3,7-dihydroimidazo [1,2-a] pyrazin-3-one hydrochloride (MCLA) was purchased from Tokyo Kasei (Tokyo, Japan). Superoxide dismutase (SOD) and sodium hypochlorite solution were purchased from Wako Pure Chemical Industries (Osaka, Japan). Aminophenyl fluorescein (APF) and hydroxyphenyl fluorescein (HPF) were purchased from GORYO Chemical (Sapporo, Japan).

fMLP (1 mM) and PMA (0.1 mM) were prepared by dissolving into DMSO and stored at -80°C as stock solutions. They were diluted with Ringer-Hepes buffer (RH buffer: 154 mM NaCl, 5.6 mM KCl, and 10 mM Hepes, pH7.4) just prior to use: fMLP [1:3 (v/v)] and PMA [1:20 (v/v)]. ABAH, SOD and hypoxanthine were dissolved in RH buffer. Stock MCLA was dissolved in Milli-Q water and its concentration was adjusted according to the molar extinction coefficient after filtration.

### Whole blood collection

Blood were collected from the fingertips of volunteers using a lancet (Becton, Dickinson and Company, Franklin Lakes, NJ, USA or Nipro, Osaka, Japan) after informed consent was obtained. The blood were then preserved in BD Microtainer Tubes (Becton, Dickinson and Company, Franklin Lakes, NJ, USA) whose inside was coated by K_2_EDTA to prevent blood coagulation. The blood samples were kept at room temperature and used within 2 hours after collection. All experiments involving blood collection were performed in full compliance with the guidelines of the Research Ethics Committee of the Hamamatsu Photonics K.K. and approved by the same committee under the number H-86(57).

### Isolation of neutrophils from whole blood

Neutrophils were isolated from whole blood using Mono-Poly resolving medium (DS Pharma Biomedical, Osaka, Japan) according to the manufacturer’s instruction manual with slight modifications. Briefly, the volumes of both blood and the medium were scaled down. Typically, a 525 μl of anticoagulated blood was loaded on a 450 μl of Mono-Poly resolving medium in a microcentrifuge tube (Thermo Fisher Scientific, Waltham, MA, USA). The blood sample was then centrifuged at 400×g for 20 min at room temperature using a swing rotor. Since the middle white layer in the centrifuged tube contains neutrophils, it was recovered carefully, washed once with 750 μl of RH buffer, centrifuged again, and finally dissolved in cold RH buffer at 4°C.

### Neutrophil counting

Neutrophil counts in whole blood and that of the isolated cells were determined with Pentra MS CRP (Horiba, Kyoto, Japan) according to the manufacturer’s instruction. The isolated fraction from the whole blood mainly contained neutrophils (average 73.23% out of 16 experiments). We double-checked the total number of isolated neutrophils by direct microscope observation.

### Cell culture

HL-60 cells, a human acute promyelocytic leukemia cell line, obtained from American Type Culture Collection (Manassas, VA, USA) were maintained and differentiated to neutrophil-like cells with DMSO as previously described [10,11,15]. The cells thus differentiated were suspended in RH buffer and kept at 4°C until measurement. The cells were counted by microscope observation with Trypan Blue staining.

### Monitoring procedure of CL and FL

Basically, CL_MCLA_ and FL_APF_ were simultaneously monitored in the following procedure (see also S1 Table) with the newly developed system (CFL-P2200, Hamamatsu Photonics K.K., Hamamatsu, Japan):

1. RH buffer containing 0.5 μM MCLA, 10 μM APF and 1 mM CaCl_2_ was pre-incubated on a dedicated glass slide.
2. Incubate for 2 minutes at 37°C.
3. For negative control samples, adding SOD and ABAH (please see next section) to the solution on the slide.
4. Adding 1.5~3 μl of whole blood or suspension of 1× 10^4^ cells of isolated neutrophils, or 2× 10^4^ cells of cultured neutrophil-like cells to the solution.
5. Stirring the solution with gentle pipetting.
6. Setting the glass slide in the system.
7. Incubate for 30 seconds in the system.
8. Start the measurement.
9. Stimulation of the sample cells by adding fMLP or PMA automatically.
10. Simultaneous recording of CL_MCLA_ and FL_APF_.
11. Data analysis by the dedicated software and Excel. Stimulant enhancements of the signals of CL_MCLA_ and FL_APF_ were determined by calculating the peak areas under the curves (AUC) [10,11].

### Modifying the concentrations of O_2_^-•^ and OCl^-^ in blood samples

SOD (9 μg/mL) was used as a scavenger of O_2_^-•^ [16–18] and ABAH (100 μM) was used as an MPO specific inhibitor [19,20]. O_2_^-•^ was generated by adding xanthine oxidase (67 units) into blood samples containing hypoxanthine (0, 0.1 and 0.2 nM) [11]. OCl- was increased by injecting an appropriate amount of sodium hypochlorite solutions (6 different concentrations) to blood samples.

### Optical configuration of the system

**Fig 1:**
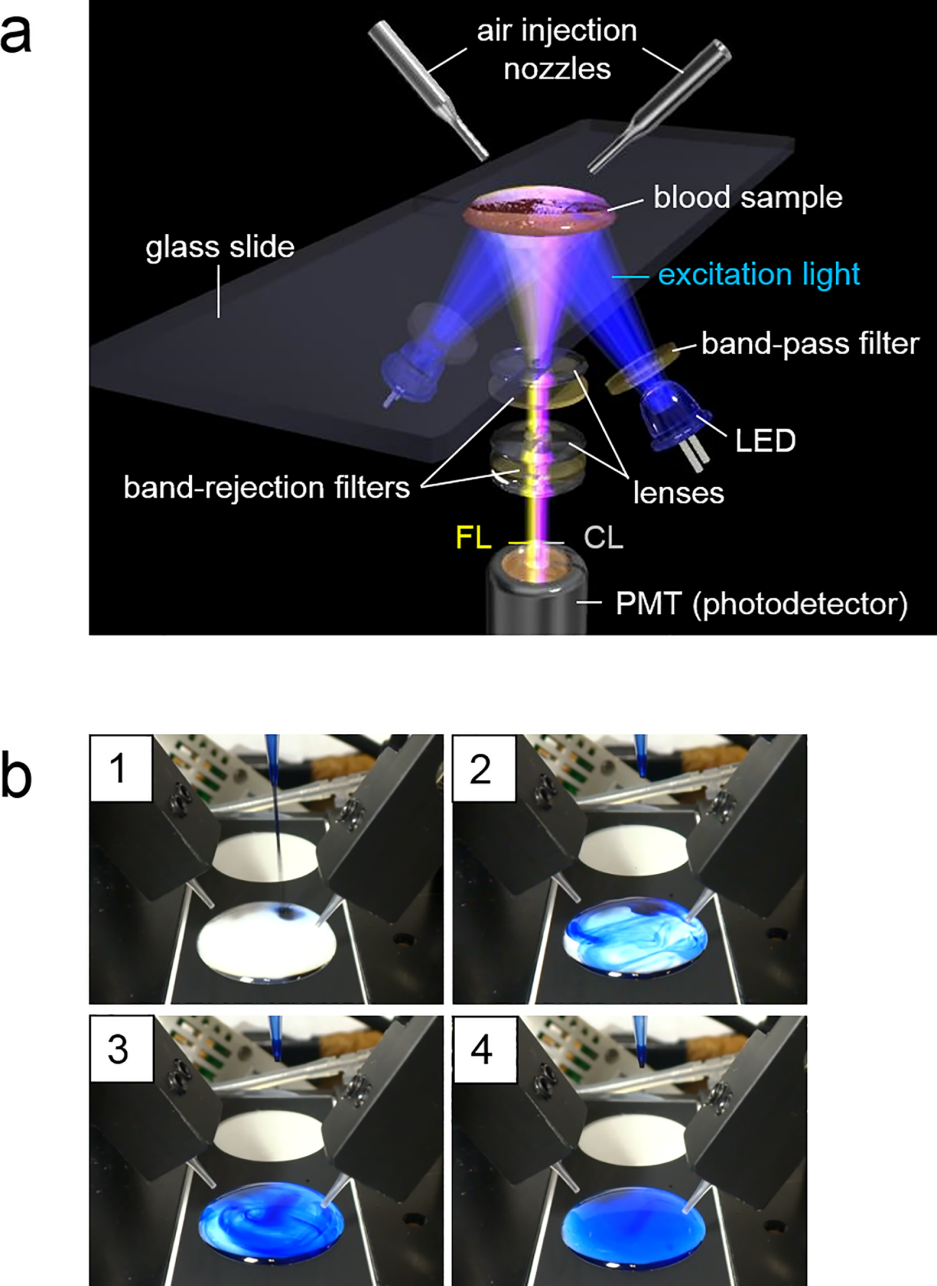
The measuring system to detect CL_MCLA_ and FL_APF_ in a tiny droplet of whole blood. (a) Schematic diagram of CFL-P2200. Detailed explanations of the system are described in the text. Dr. Hiroshi Satozono (Hamamatsu Photonics K.K.) helped us create this image. (b) Visualization of the stirring process by air flows using blue ink. Panels 1–4 show how dropped ink in liquid on a glass slide was diffused. The diffusion of ink was completed in about 2 seconds (panel 4, see also S1 Movie).

CL_MCLA_ and FL_APF_ were both detected by using a single photomultiplier tube (PMT; H10682-210, Hamamatsu Photonics K.K.; Fig 1A), with 480 nm excitation light (band-pass filter 480 nm, FWHM 10 nm), which is optimized for the FL reagent APF (Ex-Max 490nm). Further, we placed light emitting diodes (LED) as the excitation lights at the photodetector side (Fig 1A and Panel b in S1 Fig). Same as the previous system [10,11], the lens set was arranged in front of the PMT (Fig 1A). This lens set transmits FL_APF_ (Em-Max 515 nm) and CL_MCLA_ (Em-Max 465 nm). The band rejection filters (Fig 1A) were optimized for removing excitation light efficiently. The signal separation between FL_APF_ and CL_MCLA_ by using a single PMT was attained by repeating on/off of the excitation lights at a high speed, which was already used in the previous system [10,11].

### Blood sample holder

In order to ensure the stability of sample droplet shapes, the glass slide used in the newly developed CFL-P2200 has two circular areas (19 mm diameter) with printed rims made of a highly water-repellent material (a custom product by Matsunami Glass Ind., Ltd.). Fig 1A and Panel b in S1 Fig show that sample mixtures containing blood were spread over the predetermined areas on it. Since the signal crosstalks were suppressed effectively, two adjacent samples can be measured simultaneously (e.g., measuring a test and a control samples).

### Prevention of evaporation from thin sample suspensions set in the system chamber

Stirring and mixing are necessary for adding stimulants or for preventing blood cells from aggregation, but placing a magnetic bar into the thin sample suspensions in the system may disrupt the thin optical paths and interfere with the optical detection from below. So, as a means of mixing, we adopted air flows with the air-injecting nozzles placed above the samples (Fig 1A). For keeping the sample temperature at 37°C and preventing evaporation in thin samples under constant air flows, we used warmed air flows and installed a humidifier inside the sample holding chamber (100% humidity). The chamber was covered with rubber heaters which can automatically control the chamber temperature.

## Results

### Comparison of the cuvette-type cell and the thin suspensions on a glass slide

The previous system (CFL-C2000) [10,11] used a cuvette (10 mm square) as the sample container (Panel a in S1 Fig). However, such cuvettes loaded with colored samples containing light absorbing substances such as blood are susceptible to strong light absorptions. To circumvent this problem, we selected a glass slide as the sample holder in the new system (Panel b in S1 Fig). The optical path length of a sample placed on it becomes about one fourth shorter than that of the cuvettes.

To find out the improvements in optical signal strengths we compared the CL_MCLA_ data obtained with the previous (CFL-C2000, cuvette-type) and the new (CFL-P2200, glass slide-type) systems. The highest peak area of the CL_MCLA_ signals was obtained with 750-fold dilution of whole blood in cuvettes and the corresponding data of the samples on a glass slide was obtained with 500-fold dilution.

### Effects of air-flow stirring on solution mixing and CL/FL signals

For the air-flow stirring to work effectively and for obtaining a stable light signals, we carefully adjusted the amounts, the timings and the directions of the air flows. When the air flows were too strong, the FL_APF_ signal intensity fluctuated noticeably due to liquid surface disturbances. Conversely, absence of air flows or unsuitable directions resulted in erroneous measurements, due to insufficient diffusion of the added stimulant and blood cell aggregations (Panel c in S2 Fig). For fine-tuning the stirring efficiency, blue ink was used instead of the colorless reagents. The ink dropped into a sample liquid on a glass slide diffused evenly in about 2 seconds (Fig 1B and S1 Movie), indicating that the actual stimulants dropped into blood samples are mixed efficiently in a few seconds. Panel b in S2 Fig shows an outcome of the sample mixtures containing blood on a glass slide after a measurement, suggesting that they were sufficiently stirred by the air flows.

### Selectivity of MCLA, APF and HPF for monitoring O_2_^-•^ and OCl^-^

We used MCLA as a probe to measure extra-cellular O_2_^-•^ generated by NADPH oxidase in the cell membrane as previously reported [10]. MCLA is known to have a weak nonspecific luminescence derived from auto-oxidation with phosphate buffer [21]. To minimize the auto-oxidation, we used RH buffer in this system. For OCl^-^ detection, our first choice was APF, which is a fluorescence probe monitoring some of the highly reactive ROS (hROS) in a cumulative way. APF selectively and dose-dependently releases a strongly fluorescent compound, fluorescein, upon oxidation by hROS such as ^•^OH, ONOO^-^ and OCl^-^, but not by other ROS [14]. Another similar fluorescence probe HPF, which also releases fluorescein upon oxidation by ^•^OH, ONOO^-^, on the other hand, is not reactive to OCl^-^ [14]. Therefore, OCl^-^ can be specifically detected by using HPF and APF together [14]. We compared FL_APF_ and FL_HPF_ from isolated neutrophils. S3 Fig shows the net increases of both FL_HPF_ (ΔHPF) and FL_APF_ (ΔAPF), which are FL subtractions of probe only in vehicle from probe in PMA-stimulated neutrophils. The FL increase of ΔHPF was less than that of ΔAPF and AUC of the ΔHPF was 90.7% less than that of the ΔAPF.

**Fig 2:**
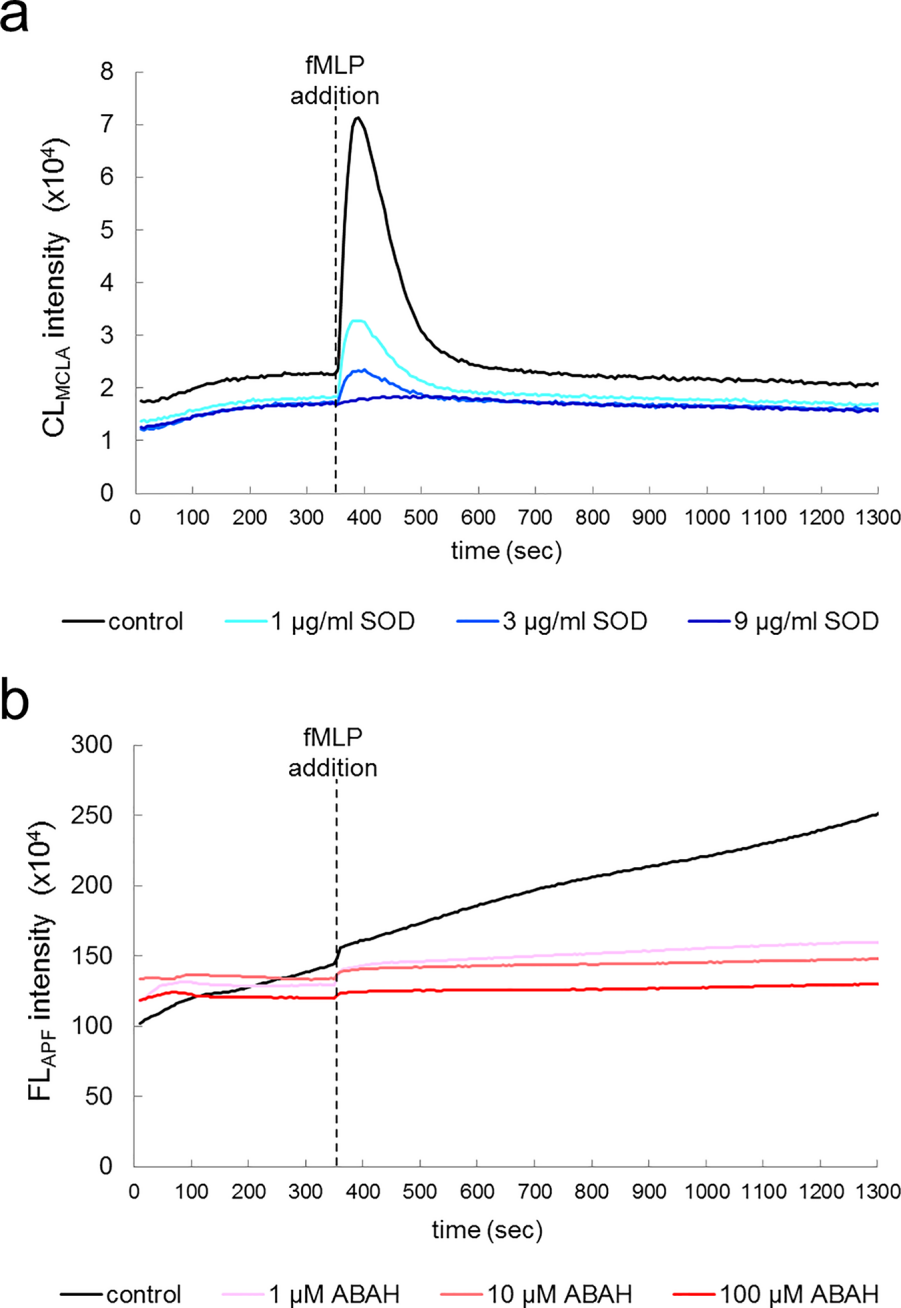
Measurements of CL_MCLA_ and FL_APF_ in neutrophil-like cells. Time courses of CL_MCLA_ (a) and FL_APF_ (b) from neutrophil-like cells. CFL-P2200 was used for the measurements. The cell mixtures were treated with control vehicle (black lines) or different concentrations of SOD (scavenger of O_2_^-•^, blue lines) and ABAH (inhibitor of MPO, red lines). fMLP was added at 350 second (dotted lines).

Next we measured CL_MCLA_ and FL_APF_ from neutrophil-like cells derived from cultured HL-60. Fig 2A shows that, soon after the stimulation (dotted line), CL_MCLA_ intensity was instantly increased and then immediately returned to the baseline level (Fig 2A, black line). This pattern was almost the same as that previously described [10,11]. To find out whether or not the signal is attributable to the generated O_2_^-•^, we scavenged O_2_^-•^ by adding SOD. Addition of three different concentrations of SOD significantly decreased the CL_MCLA_ intensity in a dose-dependent manner (Fig 2A, blue and similar colored lines). In contrast to the transient nature of the CL_MCLA_, the FL_APF_ intensity accumulated gradually by oxidation of APF (Fig 2B, black line). Likewise, to find out whether or not this signal is attributable to OCl^-^ produced by MPO, we added ABAH as an MPO specific inhibitor [19,20]. Addition of three different concentrations of ABAH markedly decreased the FL_APF_ intensity in a dose-dependent manner as expected (Fig 2B, red and similar colored lines).

### Measurements of CL_MCLA_ and FL_APF_ in a tiny droplet of whole blood

**Fig 3:**
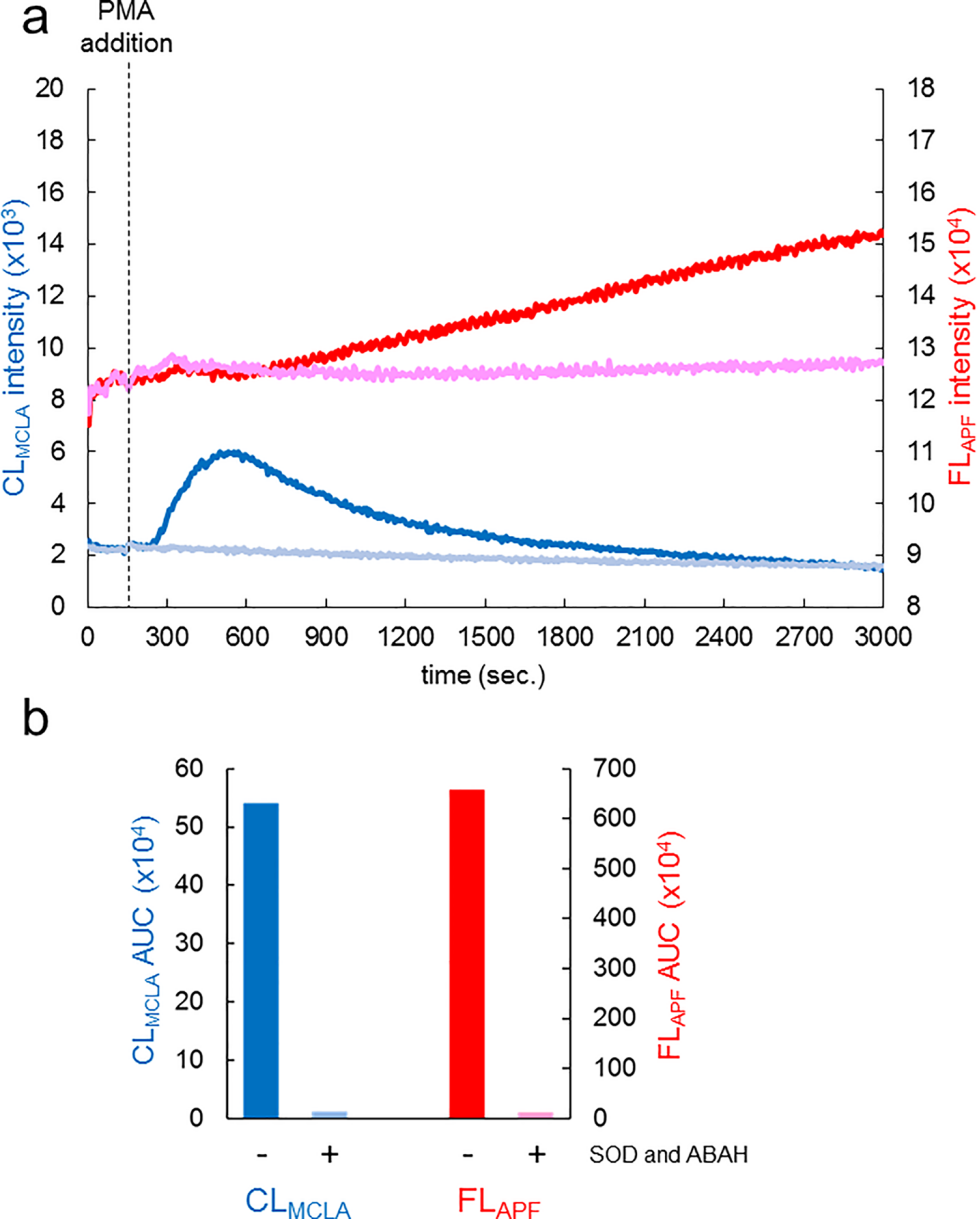
Measurements of CL_MCLA_ and FL_APF_ in whole blood. (a) Time courses of CL_MCLA_ and FL_APF_ in whole blood. CFL-P2200 was used for the measurements. The left vertical axis indicates CL_MCLA_ intensity; the right vertical axis, FL_APF_ intensity. The cell mixtures were treated with control vehicle (blue line for CL, red line for FL) or SOD and ABAH (pale blue line for CL with SOD, pink line for FL with ABAH). PMA was added at 150 second (dotted line). The baseline heights of FL_APF_ signals were adjusted for both the test sample (red line) and the sample including ABAH (pale pink line) making them start at the same level. (b) Calculated signal areas measured in (a). Increases in both CL_MCLA_ and FL_APF_ were determined by calculating AUCs.

Next we measured CL_MCLA_ and FL_APF_ elicited by a droplet of whole blood. PMA was used as a stimulant instead of fMLP because PMA was found to be more effective for stimulating neutrophils than fMLP. Blood samples were collected from a volunteer’s fingertip and kept at room temperature. They were then diluted into the reaction mixture before measurement as described in Materials and Methods and S1 Table. Fig 3A shows typical time courses of CL_MCLA_ (blue and pale blue lines) and FL_APF_ (red and pink lines), in which the left vertical axis indicates CL_MCLA_ intensity while the right vertical axis indicates FL_APF_ intensity. CL_MCLA_ slowly started to increase at around 90 seconds after PMA stimulation and then decreased gradually (Fig 3A, blue line). We could confirm that this increase corresponded to the O_2_^-•^ generated in the blood samples by observing CL_MCLA_ in the presence of SOD [16–18] (Fig 3A, pale blue line). On the other hand, the continuous increase of FL_APF_ started at around 320 seconds after PMA stimulation, which shows that FL_APF_ increase started at about the peak point of CL_MCLA_ (Fig 3A, red line). ABAH inhibition experiments confirmed that FL_APF_ was attributable to OCl^-^ [19,20]. The ABAH suppression became noticeable at about 600 seconds (Fig 3A, pale pink line). Next, we calculated AUCs of CL and FL of these experiments, which shows that AUC of CL_MCLA_ in the presence of SOD was 97.9% less than that without it (Fig 3B, left columns) and AUC of FL_APF_ in the presence of ABAH was 98.3% less than that without it (Fig 3B. right columns).

We then tested whether or not CL_MCLA_ signal can be increased when O_2_^-•^ are independently generated in blood samples by using the hypoxanthine xanthine oxidase mechanism [10,11] (Panel a in S4 Fig). When xanthine oxidase together with different concentrations of hypoxanthine was injected to the blood samples, the CL_MCLA_ signals were increased in a dose dependent manner (Panel a in S4 Fig, columns 4 and 5). Surprisingly, the notable CL_MCLA_ signal was detected when only xanthine oxidase was injected to the samples without hypoxanthine (Panel a in S4 Fig, column 3), which suggests that some amount of hypoxanthine was already in the blood samples. We also added OCl- to blood samples and confirmed that the FL_APF_ signal was proportionally increased with added OCl- concentrations and a clear linear correlation was found in the FL_APF_ signals (Panel b in S4 Fig, R = 0.998).

### Reproducibility of CL_MCLA_ and FL_APF_ measurements in blood samples

**Fig 4:**
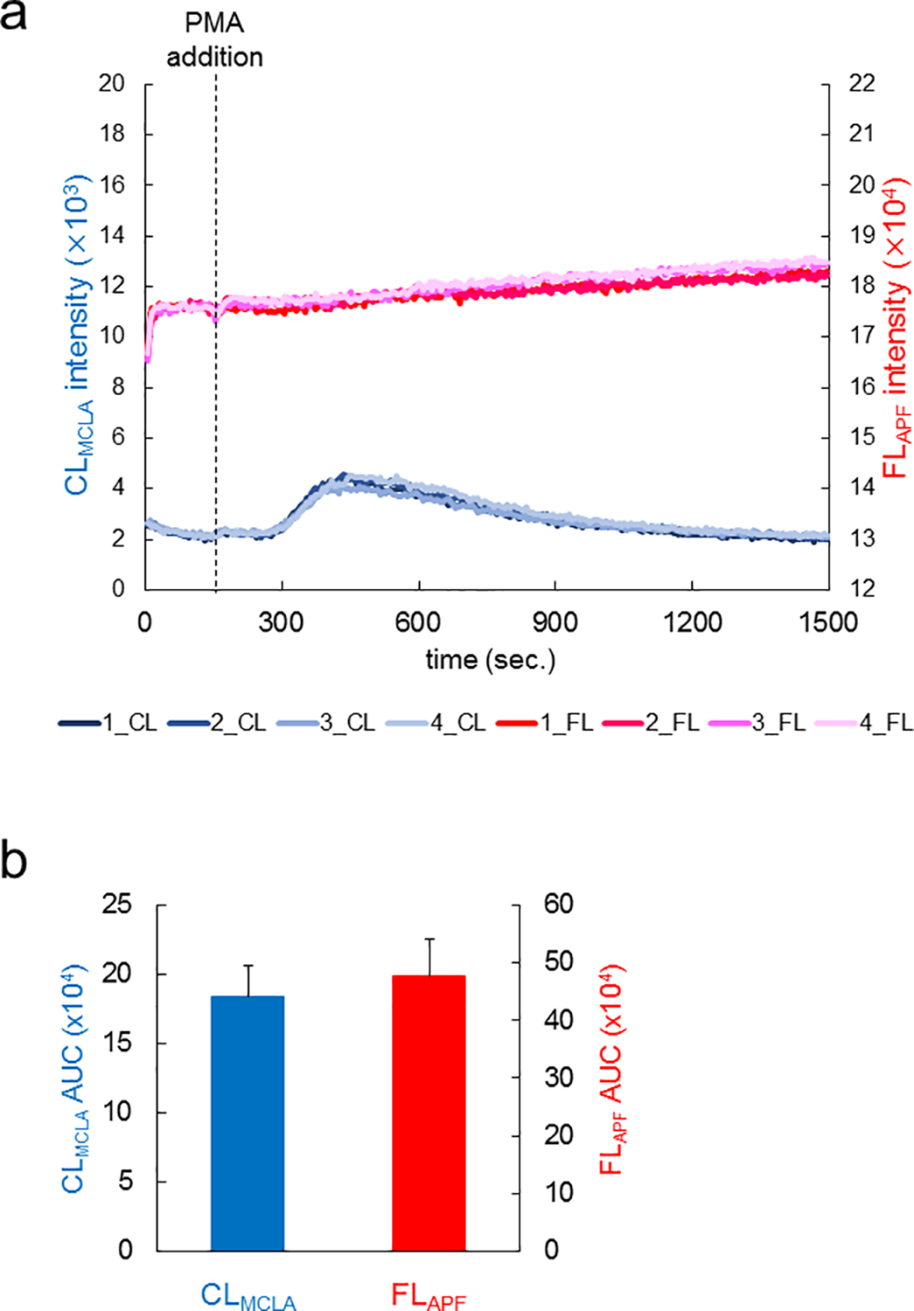
Reproducibility of CL_MCLA_ and FL_APF_ measurements in blood samples. (a) Time courses of CL_MCLA_ and FL_APF_ signals from blood samples in four independent experiments. CFL-P2200 was used for the measurements. The light colors indicate that the order of the measurement was later. The left vertical axis shows CL_MCLA_ intensity; the right vertical axis, FL_APF_ intensity. PMA was added at 150 second (dotted line). The baseline heights of FL_APF_ signals were adjusted for making the four independent experiments start at the same level. (b) Calculated signal areas measured in (a). Increases in both CL_MCLA_ and FL_APF_ were determined by calculating AUCs. Relative errors of the four measurements were 7.06% for CL_MCLA_ and 7.94% for FL_APF_.

Next we tested reproducibility of data with four different blood samples from a single volunteer preserved at room temperature in BD Microtainer Tube (see Materials and Methods; all blood samples in this experiment were measured within two hours after collection). Fig 4A shows time courses of CL_MCLA_ (blue and pale blue lines) and FL_APF_ (red and pink lines). The line color brightness indicates the order of measurement (the brightest is the latest). The reproducibility was so good that all four time courses almost overlapped with each other (Fig 4A). We calculated all four AUCs of CL_MCLA_ and FL_APF_. The relative errors of the four measurements were 7.94% for FL_APF_ and 7.06% for CL_MCLA_ (Fig 4B).

### Correlation analysis of the neutrophil data sets

**Fig 5:**
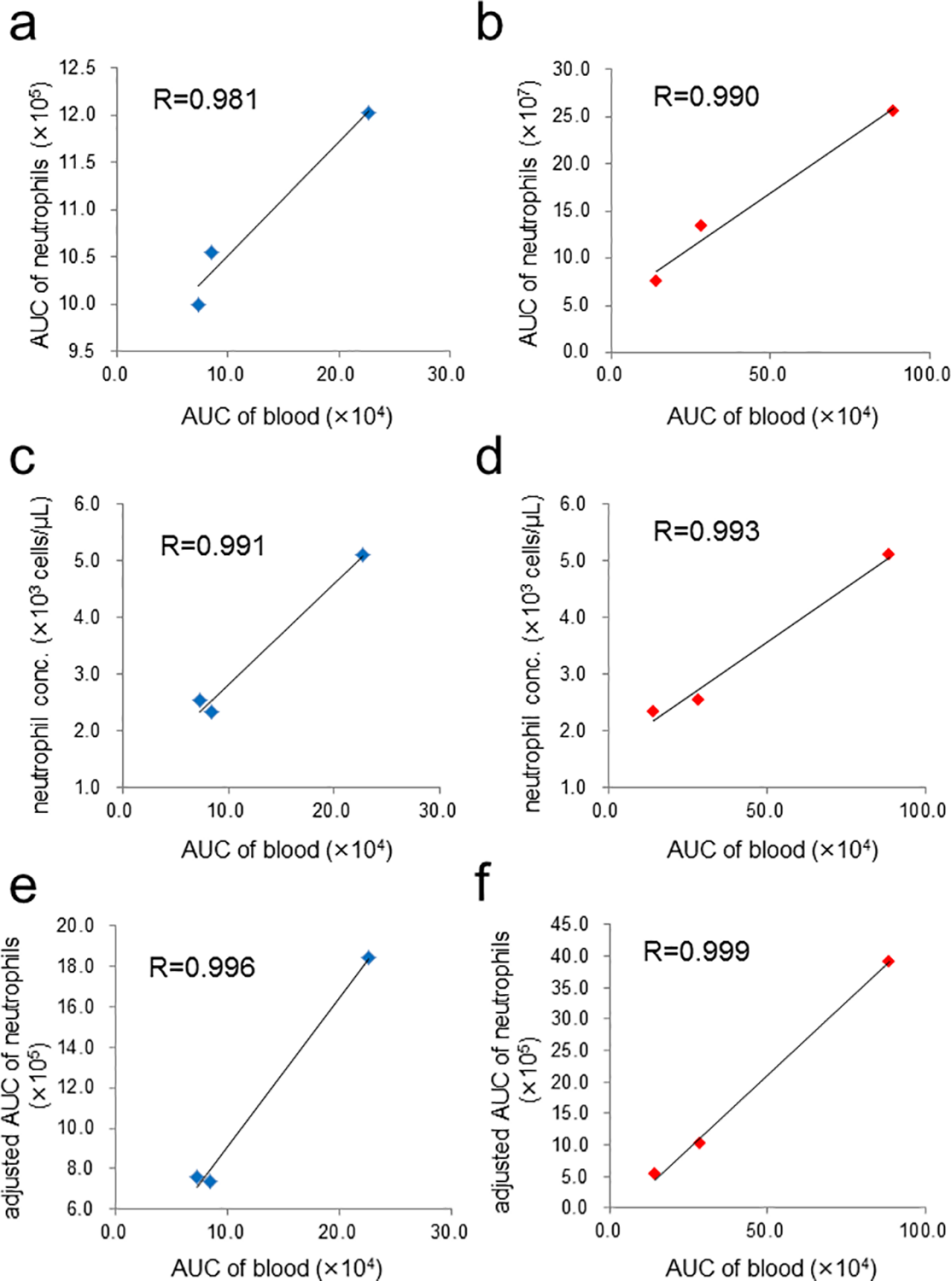
Correlations of the data sets of blood from 3 healthy volunteers. (a) Scatter plot showing a very strong positive correlation between the AUCs of CL_MCLA_ from 3 μl of whole blood (horizontal axis) and those from 1× 10^4^ cells of isolated neutrophils (vertical axis). (b) Scatter plot similarly obtained as in (a) with FL_APF_ intensities. (c) Scatter plot showing a very strong positive correlation between the AUCs of CL_MCLA_ from 3 μl of whole blood (horizontal axis) and the neutrophil concentrations in whole blood (vertical axis). (d) Scatter plot obtained as described in (c) except that the horizontal axis is the AUCs of FL_APF_ from 3 μl of whole blood. (e) Scatter plot showing a very strong positive correlation between the AUCs of CL_MCLA_ from 3 μl of whole blood (horizontal axis) and those from isolated neutrophils normalized by neutrophil counts in 3 μl of each blood (vertical axis). (f) Scatter plot obtained as described in (e) except that the horizontal axis is the AUCs of FL_APF_ intensities. CFL-P2200 was used for the monitoring of luminescence signals and Pentra MS CRP was used for measuring neutrophil concentrations in blood.

We then performed correlation analysis of CL_MCLA_ and FL_APF_ of whole blood samples from three healthy volunteers. The analysis was focused on the following three perspectives, (1) intensity (the area of increased CL_MCLA_ or FL_APF_) obtained from the whole blood samples, (2) intensity obtained from isolated neutrophils in the blood samples and (3) the concentration of the neutrophils in them. Blood samples from the three healthy volunteers were collected after fasting for 12 hours and then neutrophils were isolated as described in Materials and Methods. Neutrophil concentrations in the blood samples were determined by the conventional method (see Materials and Methods). First, we tested correlations between the whole blood samples and the isolated neutrophils. We measured CL_MCLA_ and FL_APF_ using the same amount of the whole blood (3 μl) or the same number of isolated neutrophils (1× 10^4^ cells) obtained from the volunteers. Fig 5A and 5B show their scatter plots, which shows correlations between the intensities obtained from the whole blood (horizontal axis) and those from the isolated neutrophils (vertical axis). Clear linear correlations were found in both CL_MCLA_ (Fig 5A, R = 0.981) and FL_APF_ (Fig 5B, R = 0.990). We also examined whether or not the intensities obtained from the whole blood samples were correlated with the neutrophil concentrations in each of them. Fig 5C and 5D show scatter plots which indicate correlations between the intensities obtained from the whole blood (horizontal axis) and the neutrophil concentrations in each blood (vertical axis). Again clear linear correlations were found in both CL_MCLA_ (Fig 5C, R = 0.991) and FL_APF_ (Fig 5D, R = 0.993). Then, by measuring neutrophil concentrations in the blood samples, we adjusted the intensities obtained from 1× 10^4^ cells of the isolated neutrophils (vertical axes in Fig 5A and 5B) to those from the corresponding cell counts in each blood sample (vertical axes in Fig 5E and 5F). Evidently, the most predominant correlations were found in both CL_MCLA_ (Fig 5E, R = 0.996) and FL_APF_ (Fig 5F, R = 0.999).

Finally, we supplemented the blood samples with increasing concentrations of freshly isolated neutrophils to find out whether or not the signals of CL_MCLA_ and FL_APF_ are linear across different neutrophil concentrations (S5 Fig). If there are potential interfering factors in the blood samples, linear responses of isolated neutrophils will not be obtained in our measurement. The result shows that there are clear linear correlations in both CL_MCLA_ (S5 Fig, R = 0.995) and FL_APF_ (S5 Fig, R = 0.970).

### The effects of diet and exercise on CL_MCLA_ and FL_APF_ of a tiny amount of whole blood: A pilot study of oxidative stress in volunteers

The ROS/hROS dual monitoring system we have developed may be applicable to evaluations of various factors affecting the O_2_^-•^ generation and the MPO activity in blood. To address this possibility, we studied the effect of diet and exercise on ROS/hROS in volunteers’ blood. Foods and supplements containing anti-oxidative compounds are expected to countervail ROS induced by over-activation in WBC [10,11,22,23]. In addition, the activity of MPO is known to be affected by various factors including exercise [24–27], aging [28,29], alcohol [30] and smoking [31]. We chose diet (i.e., food intake) and exercise among them since these are regarded as two of the most influential factors in our daily life.

First, we compared ROS (CL_MCLA_) and hROS (FL_APF_) before and after diet for 5 consecutive days. The blood sampling times were (1) at the end of fasting for more than 12 hours (around 7:30) and (2) within an hour after lunch (around 13:00). Lunch menus were not fixed during the experiment. Panels a (the end of fasting) and b (after lunch) in S6 Fig show day-to-day variations of CL_MCLA_ (blue), the FL_APF_ (red) and the neutrophil concentration (green). Panels c and d in S6 Fig show scatter plots which indicate correlations between the intensity of CL_MCLA_ or FL_APF_ (horizontal axis, blue: CL_MCLA_, red: FL_APF_) and the neutrophil concentration in blood (vertical axis). Fasting resulted in significant linear correlations in both CL_MCLA_ (R = 0.866) and FL_APF_ (R = 0.953) (Panel c in S6 Fig). In contrast, just after food intakes correlations became slightly poor in both CL_MCLA_ (R = 0.412) and FL_APF_ (R = 0.634) (Panel d in S6 Fig). To confirm the reproducibility of the decrease in the correlation coefficients, we conducted a similar analysis with another healthy volunteer for arbitrary 8 days (within 3 weeks). The blood sampling times were (1) at the end of fasting for more than 12 hours (around 10:00) and (2) within an hour after lunch (around 13:00). Similarly, after food intake, significant linear correlations decreased in both CL_MCLA_ (R = 0.856 to 0.477) and FL_APF_ (R = 0.844 to 0.512) (Panels e and f in S6 Fig).

Next we tested the blood ROS/hROS just before and just after exercise (within 10 minutes) for 5 consecutive days. This experiment was carried out in a hunger condition (more than 12 hours of fasting). At about the same time during these 5 days, the volunteer rode a bicycle, as exercise, in the same route for about 20 minutes. The cardiac rate and the blood pressure always showed higher values just after exercise. S7 Fig shows correlations between the intensity of CL_MCLA_ or FL_APF_ and the neutrophil concentration in blood. Before exercise, significant linear correlations were noticed in both CL_MCLA_ (R = 0.866) and FL_APF_ (R = 0.953) (Panel a in S7 Fig). After exercise, correlations decreased in both CL_MCLA_ (R = 0.584) and FL_APF_ (R = 0.150) (Panel b in S7 Fig).

## Discussion

By retaining the optical configuration of the previous ROS/intracellular Ca^2+^ dual monitoring system (CFL-C2000) [10,11] and by improving all other parts of the system, we have developed a new system aiming at measuring the oxidative stress in whole blood easily and accurately. For fulfilling that purpose, the new system is optimized for simultaneously detecting ROS and hROS, elicited mostly by WBC in a tiny amount of blood. Moreover, the system is now able to measure those signals stably for a very long time by an efficient air-flow stirring without evaporating blood samples.

The CL probe, MCLA, is commonly used for detecting one of ROS, O_2_^-•^ [12,13]. It is known to be auto-oxidized and be releasing a nonspecific weak CL, which is not inhibited by SOD [16,18]. It is also known to be reacting with singlet oxygen (^1^O_2_), which is not scavenged by SOD [17]. The FL probes, APF and HPF, are also widely used for detecting hROS [14]. APF is known to be oxidized by OCl^-^, hydroxyl radical (^•^OH) and peroxynitrite (ONOO^-^), releasing a fluorescent molecule fluorescein, while HPF is only oxidized by ^•^OH and ONOO^-^, releasing the same molecule, fluorescein [14]. Therefore, OCl^-^ can be detected by APF and HPF in combination [14].

Our results obtained by this new ROS/hROS dual monitoring system (CFL-P2200) show at least four important findings. First, the experiments on modifying O_2_^-•^ concentrations (Figs 2A and 3A and Panel a in S4 Fig) showed that CL_MCLA_ corresponded to O_2_^-•^. In particular, the finding that O_2_^-•^ scavenging by SOD inhibited most of CL_MCLA_ clearly shows that ROS generated by neutrophils, neutrophil-like cultured cells and whole blood were mostly O_2_^-•^.

Second, the two types of stimulants, fMLP and PMA, activated those cells in quite different time courses (Figs 2A and 3A). The start time of O_2_^-•^ releasing from PMA-stimulated cells was more delayed than that from fMLP. Also, the O_2_^-•^ generating period of PMA-stimulated cells was much longer than that of fMLP. A probable explanation is that these stimulants differently affected protein kinase C, which in turn activated NADPH oxidase for O_2_^-•^ generation [32, 33].

Third, we found that ABAH decreased FL_APF_ in isolated neutrophils (Fig 2B) and sodium hypochlorite solution increased FL_APF_ (Panel b in S4 Fig) in dose dependent manners. In addition, the increase of FL_HPF_ was considerably smaller than that of FL_APF_ (S3 Fig). These results show that the majority of hROS generated by isolated neutrophils were OCl^-^. Even when whole blood samples were used, the time derivative of FL_APF_ revealed that the increase of FL_APF_ began later than that of CL_MCLA_ (Fig 3A) and ABAH appreciably inhibited FL_APF_ (Fig 3)_,_ whereas FL_HPF_ was not inhibited by ABAH (data not shown). This shows that even in whole blood, FL_APF_ was mostly derived from OCl^-^ produced by MPO. However, in the case of blood, there may be more factors contributing to FL_APF_, such as yet unidentified hROS sources or some unknown artifacts.

Fourth, the generation of hROS, mostly OCl^-^, was found to be a slower process than the O_2_^-•^ generation. Since instantaneous hROS generation is proportional to the time derivative of FL (i.e., dFL/dt), the dFL_APF_/dt curve of neutrophil-like cells in Fig 2B clarified that the hROS generation started at about 400 seconds after stimulation (data not shown), and it is later than the peak of the O_2_^-•^ generation which was clearly before 400 seconds (CL_MCLA_ curve in Fig 2A). This may be explained by the fact that OCl^-^ is a product of the reaction of O_2_^-•^ catalyzed by MPO.

Since the results obtained with whole blood seem to be more complicated than those obtained with isolated cells, we tried to identify the responsible blood cells generating O_2_^-•^ and OCl^-^. Results of Fig 5 suggest that the luminescence signals were derived largely from the neutrophils in whole blood we used so far. In fact, both CL_MCLA_ and FL_APF_ were proportional to the counts of neutrophils added to the blood samples (S5 Fig), which enabled us to estimate the CL/FL intensity attributable to the neutrophil count. This was found to be about 90% of the total CL/FL observed. The residual 10% of CL/FL not attributable to neutrophils may include not yet identified hROS sources and/or some unknown artifacts.

Although measuring WBC counts has become quite easy by using commercially available devices, such counts cannot directly reveal ROS generation. Many reports suggested that excess amount of ROS by over-activated WBC are linked to various disorders [4,5]. It is also reported that ROS generation can be modified by some factors such as diet and exercise [10,11,24–27], which was also shown by our results (S6 and S7 Fig). Therefore, it would be very important to develop a method for detecting ROS directly. For this purpose, isolated neutrophils have so far been used in many studies [24]. However, neutrophil isolation requires several steps and at least a few milliliters of blood. In addition, the procedures might cause stress on neutrophils prior to measurement. Moreover, behavior of neutrophils in blood might be different from that of isolated neutrophils. Since the present system can measure ROS/hROS in whole blood, these drawbacks would be eliminated. Further, it may be able to monitor other types of cells (such as monocytes) in situations caused by some diseases [34]. In order to prevent such diseases caused by oxidative stress and improve individual physical conditions, it would also be important to detect an imbalance of oxidative states *in vivo* at early stages.

As the new system enabled O_2_^-•^/OCl^-^ dual monitoring in whole blood, we conducted a pilot study on the effects of diet and exercise on generated O_2_^-•^ and OCl^-^ as indicators of oxidative stress. The results show that the correlations between the ROS generation and the neutrophil concentrations were decreased after diet (S6 Fig) and exercise (S7 Fig), which may suggest that the neutrophil counts cannot reveal the whole picture of the oxidative stress. Therefore, the present system may be applicable to cases in which neutrophil counts do not change appreciably. Results in S6 Fig may indicate that some kinds of functional compounds in food interacted with the oxidative substances contained in the blood and/or modulated the O_2_^-•^/OCl^-^ generation of circulating neutrophils. Results in S7 Fig may also suggest that exercise modulated O_2_^-•^/OCl^-^ generation of circulating neutrophils [26] and/or induced enhancement of plasma antioxidant activity [27], depending on a day-to-day variance in the physical conditions. Further studies by increased number of subjects, fixed diet/quantitated exercise are important in the future.

Recently, various biomarkers for excess activities of MPO were developed [35–37]. They are good indicators to disease symptoms, which can be regarded as “products” of oxidative stress. On the other hand, what our system can evaluate is ROS and hROS generation, which can be regarded as “substrates” of oxidative stress. Therefore, combining such biomarkers and the present system may reveal the mechanisms and processes of oxidative stress in more detail. We are also planning to clarify detailed relationships between some disorders and ROS/hROS generation.

Another advantage of this system is that it is readily designed to monitor not only blood samples but also various types of cells including adhesive cells. Although we were able to measure O_2_^-•^ generation from floated cultured neurons by using the previous cuvette-type system [38], the present system can do it more easily and effectively. Moreover, since the new system is very sensitive to the optical signals, it may also be applicable to cells not actively producing ROS, such as muscle cells and neurons. Further, this system may be applicable to measurements of more than two luminescence signals simultaneously from various suspension samples including non-biological materials. In spite of these advantages, more improvements are still possible and practical, such as auto-adjustment of air flows and automatic handling of liquids and blood samples. Therefore, we are also hoping to make it even easier to operate so that it can publicly be accessible at various facilities such as citizen centers, drug stores and gyms in order to help people select suitable anti-oxidative foods or evaluate daily physical conditions for preventing diseases.

## Supporting information

S1 Fig Schematic diagrams for comparing the measurement principles of CFL-C2000 and CFL-P2200. (a) Schematic diagrams illustrating the measurement principle of CFL-C2000. The left diagram shows the top view of the system, the right panel, a side view of the system. The system used the cuvette as a sample container for cultured neutrophil-like cells. LED (excitation light) was placed at a right angle of the photodetector on the same side. (b) Schematic diagram illustrating the measurement principle of CFL-P2200. A sample mixture containing blood was spread over a wide area on the glass slide to make the optical pass length as short as possible. Two LEDs (excitation lights) were placed at the photodetector side (luminescence signals) to minimize light absorptions in whole blood.
(TIF)

S2 Fig Actual images of blood samples on the custom ordered double-well slide glass before and after measurements. (a) Blood samples on a dedicated slide glass just before measurement. The samples containing whole blood were mixed with gentle pipetting prior to measurement. (b) Blood samples on the glass slide just after measurement using CFL-P2200. Air flows were used for diffusing stimulants dropped into the blood samples and for preventing blood cells from aggregation. (c) Blood samples on the glass slide with insufficient stirring by weaker air flows, showing that the blood components were clumped in the bottom of the wells.
(TIF)

S3 Fig Fluorescence signals of HPF or APF from suspensions of isolated neutrophils. Time courses of the fluorescence signals of HPF (blue) or APF (orange). The net increases of both FL_HPF_ (ΔHPF) and FL_APF_ (ΔAPF) obtained by subtracting the intensity of vehicle from that of PMA stimulated neutrophils were plotted. PMA or the corresponding amount of vehicle was added at 350 second (dotted line). CFL-P2200 was used for the measurements.
(TIF)

S4 Fig Selectivity of MCLA and APF using the newly developed system. (a) Column charts showing the selectivity of MCLA, which was used as a CL probe in this study. The vertical axis indicates the CL_MCLA_ AUCs and the horizontal rows, as follows: Column 1: xanthine oxidase (67 units) was injected to the sample containing no blood but 0.1 nM hypoxanthine. Column 2: RH buffer was injected to the sample containing blood but no hypoxanthine. Column 3: xanthine oxidase (67 units) was injected to the sample containing blood but no hypoxanthine. Columns 4 and 5: xanthine oxidase (67 units) was injected to the samples containing both blood and different concentrations of hypoxanthine (0.1 or 0.2 nM). (b) Scatter plot showing the selectivity of APF, which was used as an FL probe in this study. OCl^-^ was independently increased in the blood samples by injecting different concentrations of sodium hypochlorite solution (relative concentrations: ×1, ×3.3, ×10, ×33 ×100, ×330). The vertical axis indicates the FL_APF_ AUCs and the horizontal axis, relative concentrations of injected sodium hypochlorite solution. The FL_APF_ signals were increased in a dose dependent manner (R = 0.998). CFL-P2200 was used for the measurements.
(TIF)

S5 Fig Evaluation of the linearity of the signals of CL_MCLA_ and FL_APF_ across different neutrophil concentrations. Scatter plot showing that the luminescence signals were linear across different neutrophil concentrations in blood. CFL-P2200 was used for the measurements. The vertical axes indicate the CL_MCLA_ AUCs (a) and the FL_APF_ AUCs (b). Blood samples were supplemented with various counts of freshly isolated neutrophils (horizontal axis). Clear linear correlations were found in both CL_MCLA_ (R = 0.995) and FL_APF_ (R = 0.970).
(TIF)

S6 Fig Effects of diet on CL_MCLA_ and FL_APF_ in whole blood. (a) Daily variations of the CL_MCLA_ AUCs (blue), the FL_APF_ AUCs (red) and the neutrophil concentration (green) before diet. The left vertical axis indicates CL_MCLA_ and FL_APF_ AUCs. The right vertical axis indicates neutrophil concentrations in whole blood. Blood was collected from a healthy volunteer and measured at the same time on 5 consecutive days (horizontal axis). (b) Daily variations of them obtained as described in (a) after diet. (c) Superimposed scatter plots showing correlations between the AUCs of CL_MCLA_ (blue) or FL_APF_ (red) and the neutrophil concentrations in 3 μl of whole blood before diet. The original data obtained were in (a). (d) Scatter plots after diet. The data was obtained as described in (c). The original data obtained were in (b). (e) Scatter plots derived from the data of another healthy volunteer. The data were obtained as described in (c). Blood were collected and measured for arbitrary 8 days (within 3 weeks) before diet. (f) Scatter plots after diet. The data were obtained as described in (e). CFL-P2200 was used for the monitoring of luminescence signals and Pentra MS CRP was used for measuring neutrophil concentrations in blood.
(TIF)

S7 Fig Effects of exercise on CL_MCLA_ and FL_APF_ in whole blood. (a) Scatter plots showing correlations between the AUCs of CL_MCLA_ (blue) or FL_APF_ (red) and the neutrophil concentrations in 3 μl of whole blood before exercise. Blood samples were collected and measured for 5 consecutive days from a healthy volunteer. Original data were obtained in Panel a in S6 Fig. (b) Scatter plots after exercise. The data were obtained as described in (a). CFL-P2200 was used for monitoring luminescence signals and Pentra MS CRP was used for measuring neutrophil concentrations in blood.
(TIF)

S1 Table Scheme showing the procedure to measure blood samples by using CFL-P2200. The first column in the table shows the reagents in the reaction mixture and the second column shows the stock concentrations of each reagent. The reaction mixture were prepared and measured on the dedicated glass slide and obtained data were analyzed according to the steps 1–11 on the lower side in the table.
(TIF)

S1 Movie Visualization of the air-flow stirring process of the newly developed system. The movie shows how dropped blue ink into a measuring sample on the glass slide is diffused. The ink was fully diffused in about 2 seconds (see also Fig 1B).
(MP4)
