# Supplementary figures and images for: Rapid on-site dual optical system to measure specific reactive oxygen species (O2-• and OCl-) in a tiny droplet of whole blood
Source: PLoS One. 2018 Aug 1;13(8):e0200573. doi: 10.1371/journal.pone.0200573 (PMC6070198; doi:10.1371/journal.pone.0200573)

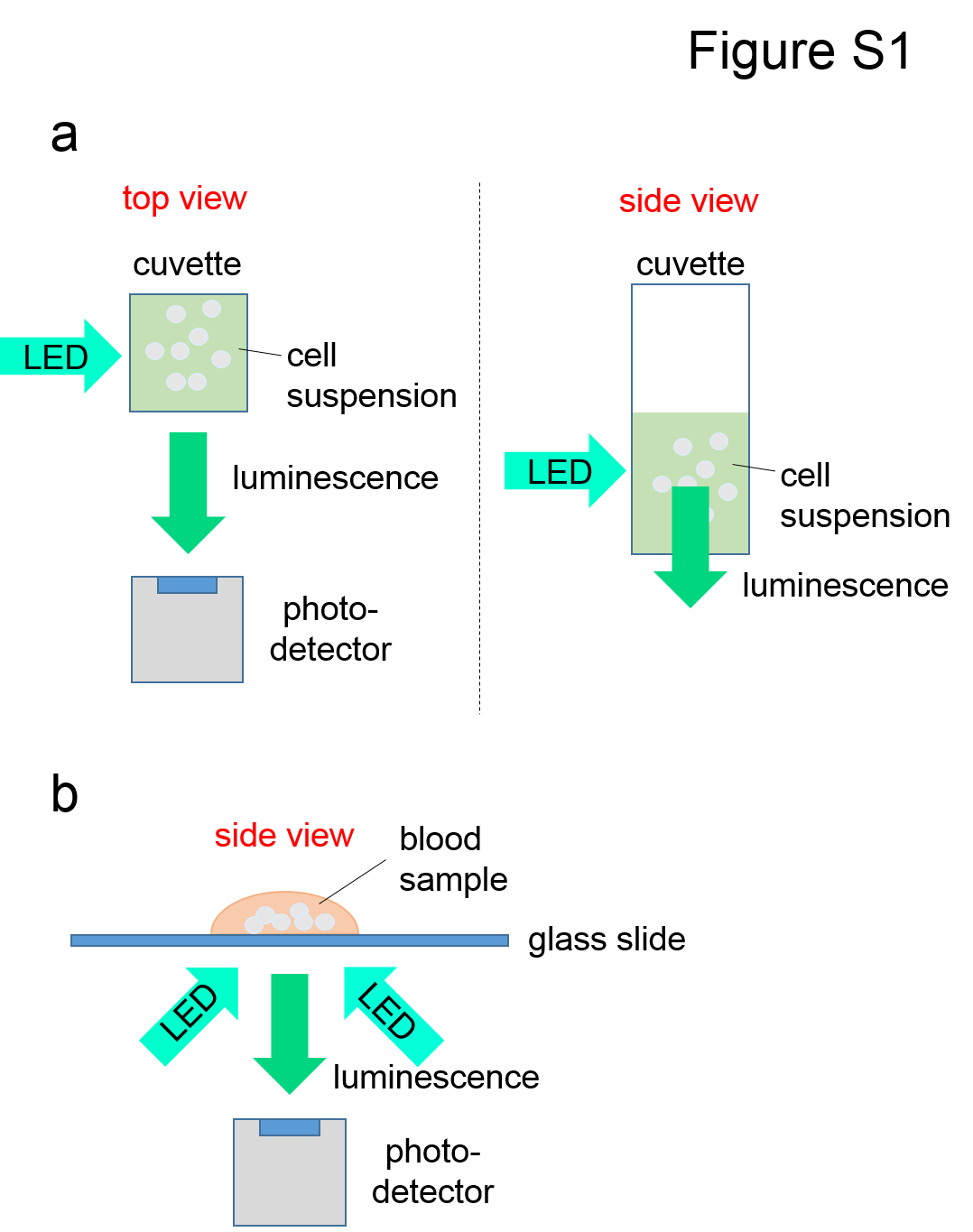

Supplement: S1 Fig — (a) Schematic diagrams illustrating the measurement principle of CFL-C2000. The left diagram shows the top view of the system, the right panel, a side view of the system. The system used the cuvette as a sample container for cultured neutrophil-like cells. LED (excitation light) was placed at a right angle of the photodetector on the same side. (b) Schematic diagram illustrating the measurement principle of CFL-P2200. A sample mixture containing blood was spread over a wide area on the glass slide to make the optical pass length as short as possible. Two LEDs (excitation lights) were placed at the photodetector side (luminescence signals) to minimize light absorptions in whole blood. (TIF) [file pone.0200573.s001.tif]

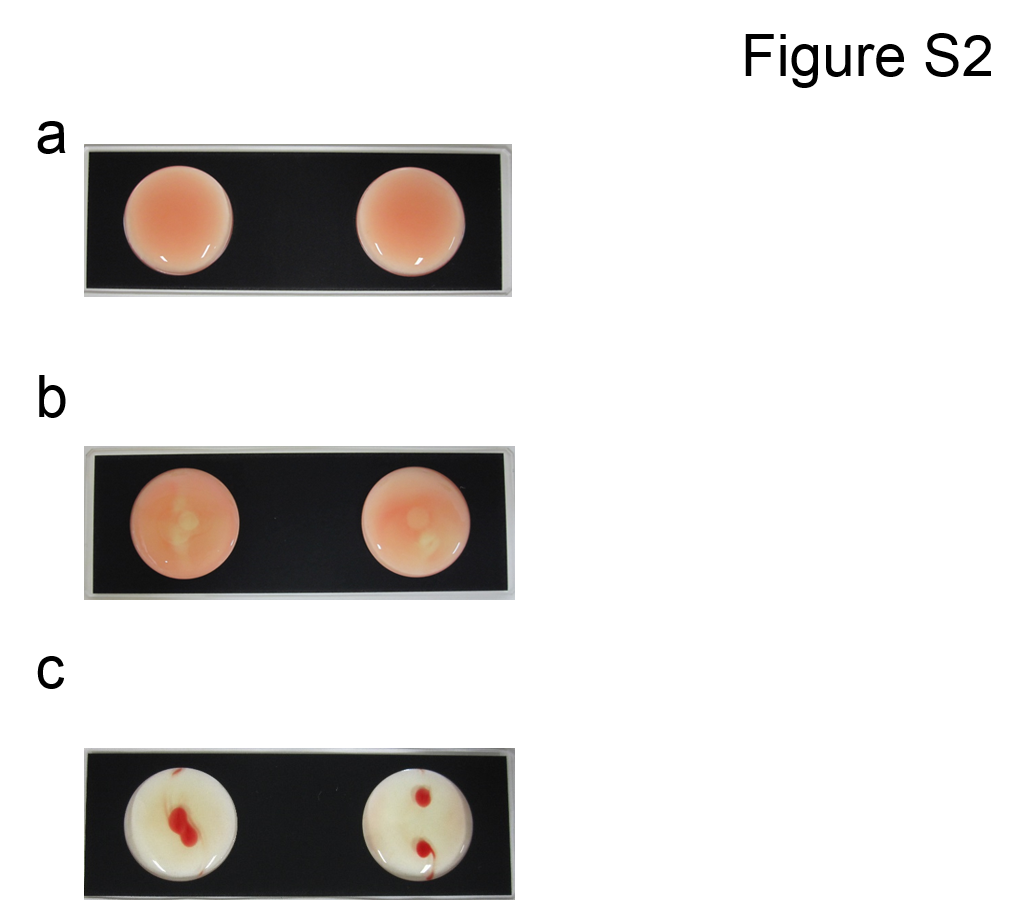

Supplement: S2 Fig — (a) Blood samples on a dedicated slide glass just before measurement. The samples containing whole blood were mixed with gentle pipetting prior to measurement. (b) Blood samples on the glass slide just after measurement using CFL-P2200. Air flows were used for diffusing stimulants dropped into the blood samples and for preventing blood cells from aggregation. (c) Blood samples on the glass slide with insufficient stirring by weaker air flows, showing that the blood components were clumped in the bottom of the wells. (TIF) [file pone.0200573.s002.tif]

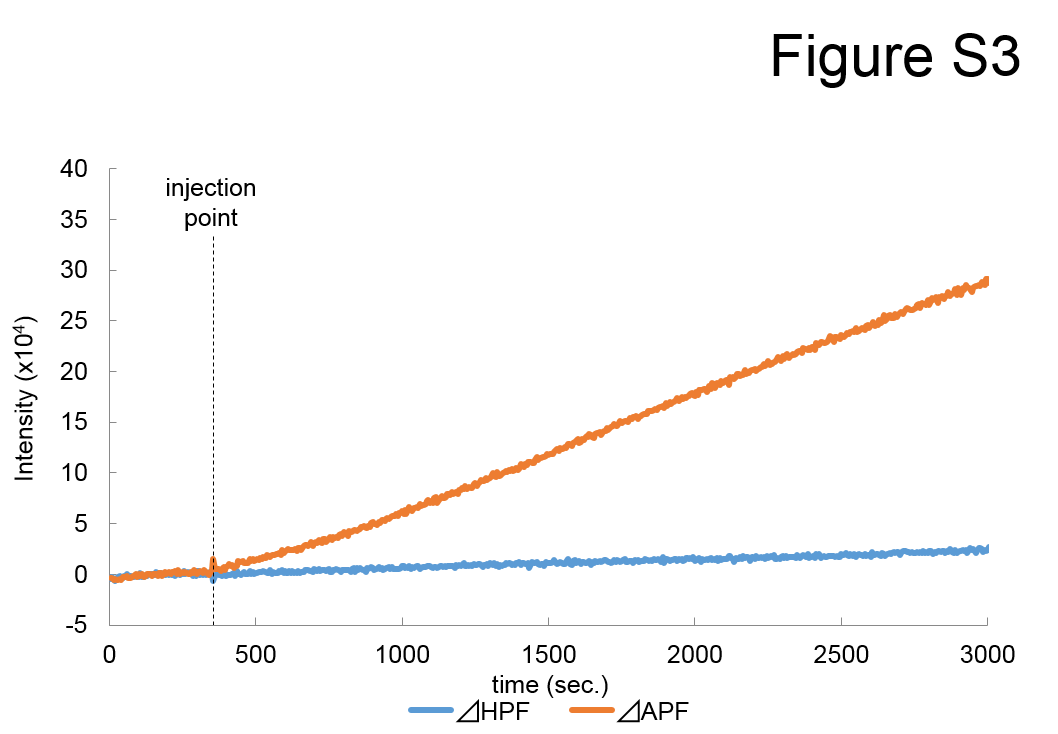

Supplement: S3 Fig — Time courses of the fluorescence signals of HPF (blue) or APF (orange). The net increases of both FLHPF (ΔHPF) and FLAPF (ΔAPF) obtained by subtracting the intensity of vehicle from that of PMA stimulated neutrophils were plotted. PMA or the corresponding amount of vehicle was added at 350 second (dotted line). CFL-P2200 was used for the measurements. (TIF) [file pone.0200573.s003.tif]

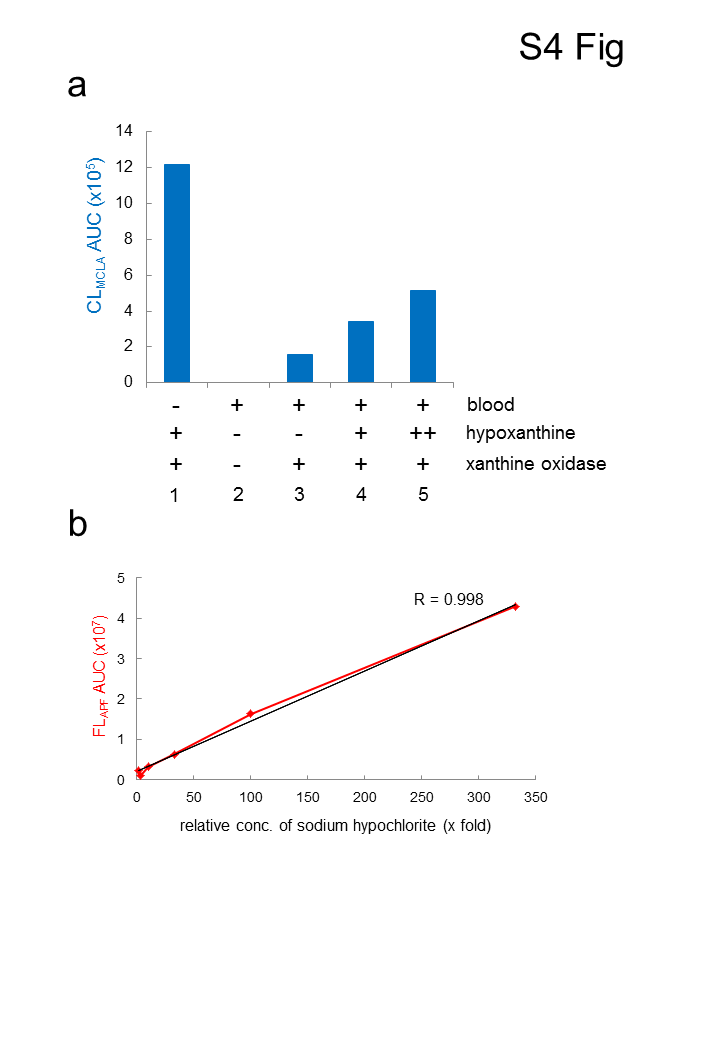

Supplement: S4 Fig — (a) Column charts showing the selectivity of MCLA, which was used as a CL probe in this study. The vertical axis indicates the CLMCLA AUCs and the horizontal rows, as follows: Column 1: xanthine oxidase (67 units) was injected to the sample containing no blood but 0.1 nM hypoxanthine. Column 2: RH buffer was injected to the sample containing blood but no hypoxanthine. Column 3: xanthine oxidase (67 units) was injected to the sample containing blood but no hypoxanthine. Columns 4 and 5: xanthine oxidase (67 units) was injected to the samples containing both blood and different concentrations of hypoxanthine (0.1 or 0.2 nM). (b) Scatter plot showing the selectivity of APF, which was used as an FL probe in this study. OCl- was independently increased in the blood samples by injecting different concentrations of sodium hypochlorite solution (relative concentrations: ×1, ×3.3, ×10, ×33 ×100, ×330). The vertical axis indicates the FLAPF AUCs and the horizontal axis, relative concentrations of injected sodium hypochlorite solution. The FLAPF signals were increased in a dose dependent manner (R = 0.998). CFL-P2200 was used for the measurements. (TIF) [file pone.0200573.s004.tif]

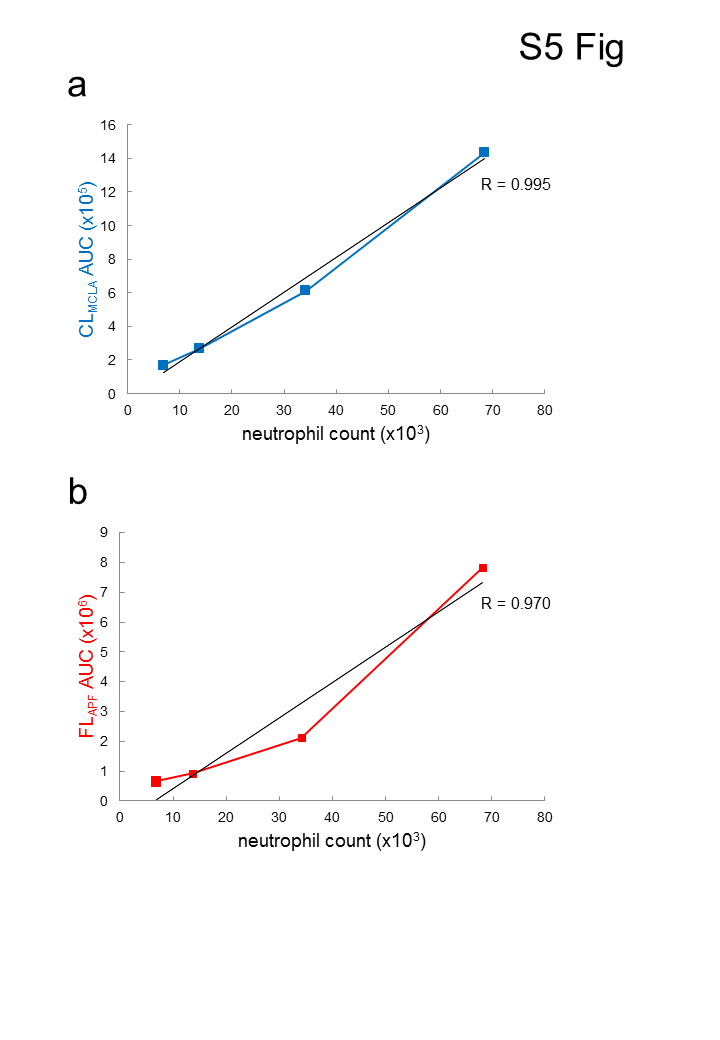

Supplement: S5 Fig — Scatter plot showing that the luminescence signals were linear across different neutrophil concentrations in blood. CFL-P2200 was used for the measurements. The vertical axes indicate the CLMCLA AUCs (a) and the FLAPF AUCs (b). Blood samples were supplemented with various counts of freshly isolated neutrophils (horizontal axis). Clear linear correlations were found in both CLMCLA (R = 0.995) and FLAPF (R = 0.970). (TIF) [file pone.0200573.s005.tif]

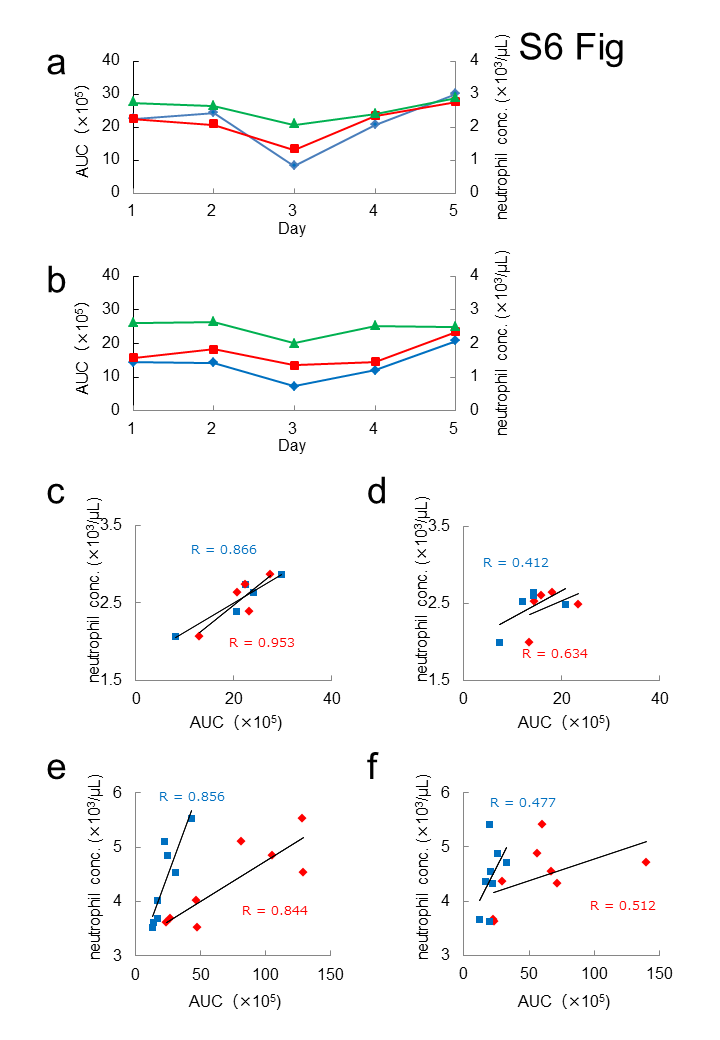

Supplement: S6 Fig — (a) Daily variations of the CLMCLA AUCs (blue), the FLAPF AUCs (red) and the neutrophil concentration (green) before diet. The left vertical axis indicates CLMCLA and FLAPF AUCs. The right vertical axis indicates neutrophil concentrations in whole blood. Blood was collected from a healthy volunteer and measured at the same time on 5 consecutive days (horizontal axis). (b) Daily variations of them obtained as described in (a) after diet. (c) Superimposed scatter plots showing correlations between the AUCs of CLMCLA (blue) or FLAPF (red) and the neutrophil concentrations in 3 μl of whole blood before diet. The original data obtained were in (a). (d) Scatter plots after diet. The data was obtained as described in (c). The original data obtained were in (b). (e) Scatter plots derived from the data of another healthy volunteer. The data were obtained as described in (c). Blood were collected and measured for arbitrary 8 days (within 3 weeks) before diet. (f) Scatter plots after diet. The data were obtained as described in (e). CFL-P2200 was used for the monitoring of luminescence signals and Pentra MS CRP was used for measuring neutrophil concentrations in blood. (TIF) [file pone.0200573.s006.tif]

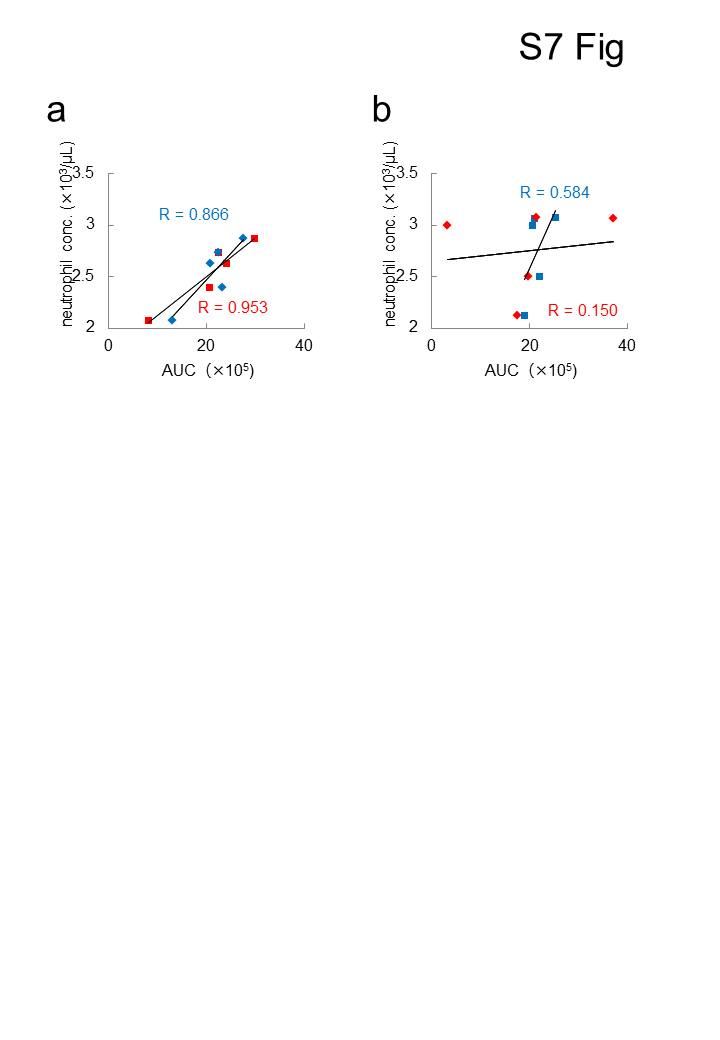

Supplement: S7 Fig — (a) Scatter plots showing correlations between the AUCs of CLMCLA (blue) or FLAPF (red) and the neutrophil concentrations in 3 μl of whole blood before exercise. Blood samples were collected and measured for 5 consecutive days from a healthy volunteer. Original data were obtained in Panel a in S6 Fig. (b) Scatter plots after exercise. The data were obtained as described in (a). CFL-P2200 was used for monitoring luminescence signals and Pentra MS CRP was used for measuring neutrophil concentrations in blood. (TIF) [file pone.0200573.s007.tif]

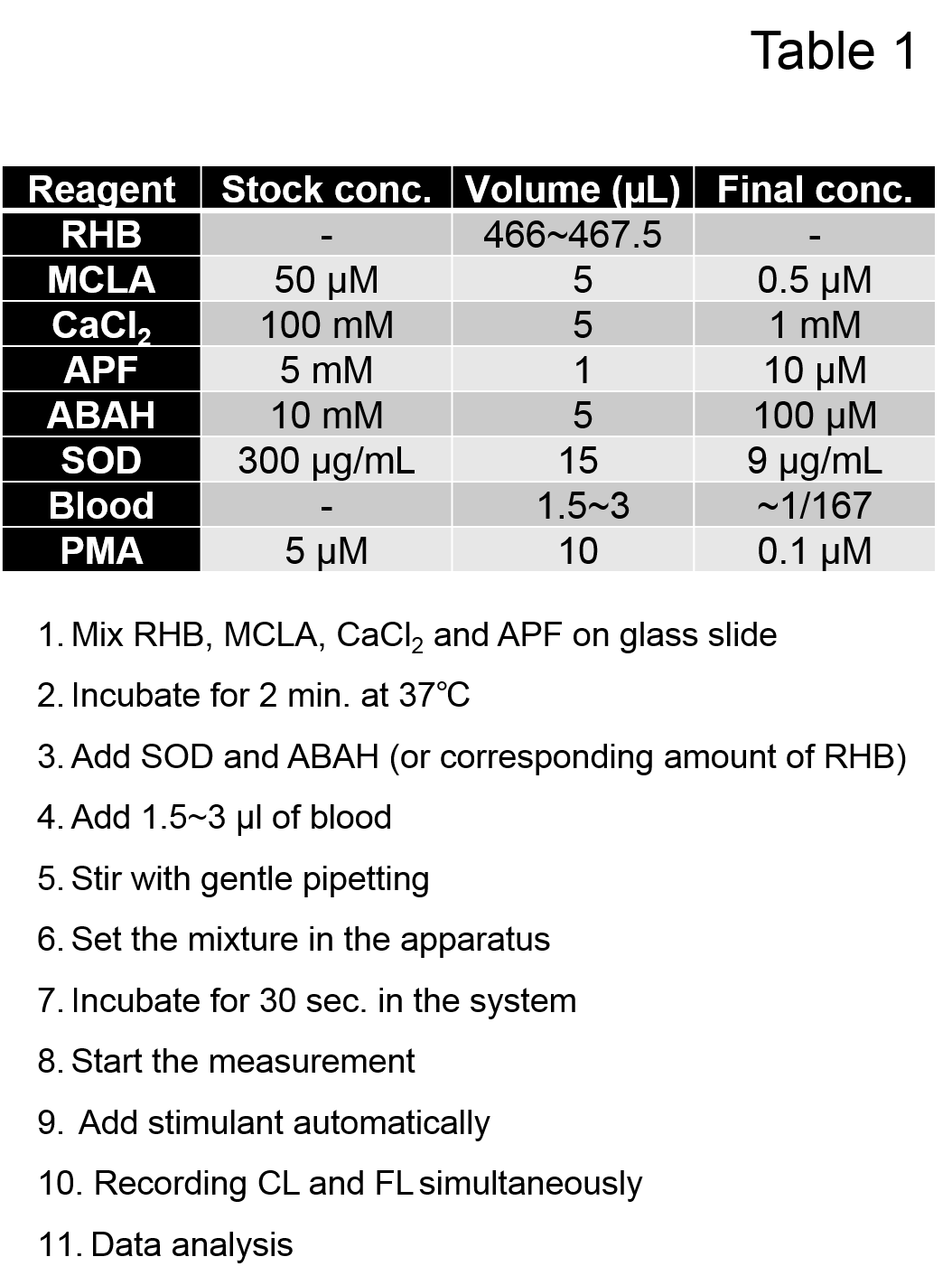

Supplement: S1 Table — The first column in the table shows the reagents in the reaction mixture and the second column shows the stock concentrations of each reagent. The reaction mixture were prepared and measured on the dedicated glass slide and obtained data were analyzed according to the steps 1–11 on the lower side in the table. (TIF) [file pone.0200573.s008.tif]
